# A patient-cohort study of numerical analysis on sacroiliac joint stress distribution in pre- and post-operative hip dysplasia

**DOI:** 10.1038/s41598-022-18752-1

**Published:** 2022-08-25

**Authors:** Ryota Toyohara, Ayumi Kaneuji, Noriyuki Takano, Daisuke Kurosawa, Niels Hammer, Toshiro Ohashi

**Affiliations:** 1 Division of Human Mechanical Systems and Design, Graduate School of Engineering, Hokkaido University, N13, W8, Kita-ku, Hokkaido, Sapporo, 060-8628 Japan; 2 Department of Orthopedic Surgery, Kanazawa Medical University, Uchinada, Japan; 3 Integrated Technology Research Center of Medical Science and Engineering, Kanazawa Institute of Technology, Nonoichi, Japan; 4 Department of Orthopedic Surgery/Japan Sacroiliac Joint and Low Back Pain Center, JCHO Sendai Hospital, Sendai, Japan; 5 Division of Macroscopic and Clinical Anatomy, Gottfried Schatz Research Center, Medical University of Graz, Graz, Austria; 6 Department of Orthopedic and Trauma Surgery, University of Leipzig, Leipzig, Germany; 7 Fraunhofer Institute for Machine Tools and Forming Technology (IWU), Medical Branch, Dresden, Germany; 8 Faculty of Engineering, Hokkaido University, Sapporo, Japan

**Keywords:** Experimental models of disease, Computational models, Bone, Bone quality and biomechanics

## Abstract

In acetabular dysplasia, the cartilaginous roof on the acetabular side does not fully cover the femoral head, which may lead to abnormal stress distribution in both the femoral head and pelvis. These stress changes may have implications to the adjacent sacroiliac joint (SIJ). The SIJ has a minimal range of motion and is closely coupled to the adjacent spine and pelvis. In consequence, the SIJ may react sensitively to changes in stress distribution at the acetabulum, with hypermobility-induced pain. The purpose of this study was to investigate the stress distribution of the SIJ in acetabular dysplasia, and to gain insight into the cause and mechanisms of hypermobility-induced pain at the SIJ. Finite element models of pre- and postoperative pelves of four patients with acetabular dysplasia were created and analyzed in double leg standing positions. The preoperative models were relatively inflare, the sacral nutation movement, SIJ cartilage equivalent stress, and the load on the surrounding ligaments decreased with increased posterior acetabular coverage. Acetabular morphology was shown to affect the SIJ, and improvement of the posterior acetabular coverage may help normalize load transmission of the pelvis and thus improve the stress environment of the SIJ in acetabular dysplasia.

## Introduction

Acetabular dysplasia is one of the most influential factors to progress to osteoarthritis (OA) of the hip joint due to insufficient bony coverage of the femoral head^1–4^. As a characteristic of acetabular dysplasia, it has been reported that the iliac bone has an inward morphological abnormality^5^. The posterior part of the iliac bone constitutes the sacroiliac joint (SIJ). In the pelvis, the SIJ has a small range of motion to work as a shock absorber between the spine and lower extremities^6,7^. In humans, the SIJ plays a crucial role in the ability to walk upright on two legs (bipedal walking)^8^, as it dissipates loads effectively. Although movement in the SIJ is limited, one of the representative movements of the SIJ is nutation and counter-nutation. This is a forward and backward rotation of the sacrum on the sagittal plane, respectively. According to the principles of manual medicine for the treatment SIJ function, the SIJ would be in the most-stable position when the sacrum is in the counter-nutation position relative to the ilium, indicating a closed-packed position of the SIJ^9^. The SIJ consists of an anterior cartilaginous region and a posterior ligamentous (syndesmotic) region^10^. Particularly, the posterior ligaments play an important role in load-bearing and transmission in the pelvis^8^. Repetitive and/or unexpected movements may cause minor subluxation of the SIJ, which has been hypothesized to lead to joint dysfunction^11^. In general, a joint dysfunction can be defined as a functional disorder of the joint without known specific cause^12^, i.e., modern medical standards including imaging equipment and surgical technology are unable to detect the morphological changes^9^. When SIJ dysfunction occurs, nerve endings, mainly in the posterior ligaments of the joint^13^, may be involved in the generation of pain as an alarming sign of SIJ dysfunction. In approximately 15%-30% of patients with lower back or gluteal pain, the dysfunctional SIJ can be identified as a cause^14,15^. The diagnosis of the pain originating from the SIJ is often delayed owing to a lack of specific imaging findings, and many patients experience chronic pain without appropriate treatment. Several clinical reports mentioned that hip disorders could affect the SIJ condition and pain^16–18^. The mechanical effects on both the cartilaginous and the ligaments of the SIJ in patients with acetabular dysplasia may differ from those in the healthy pelvis.

Peri-acetabular osteotomy is a mainstay in the surgical treatment of acetabular dysplasia to prevent the progression of OA of the hip. This surgery transects the iliac and ischial bones that are in contact with the hip joint; here the osteotomized bone is rotated outward and anteriorly to improve the bony and cartilaginous coverage of the femoral head. Acetabular osteotomy, such as rotational acetabular osteotomy and periacetabular osteotomy, has been performed and good, long-term results have been reported clinically in improving hip pain and suppressing OA progression^19–22^. It is believed that acetabular osteotomy alters stress distribution at the hip joint^23^, but is unclear to date whether mechanical changes to the SIJ will occur.

In this given study, stress distribution of the whole pelvis was analyzed using preoperative and postoperative models of the osteoligamentous pelvis of four patients who underwent unilateral spherical periacetabular osteotomy^24^.

The study aimed to investigate the stress environment of the SIJ in acetabular dysplasia. It was hypothesized that the inwardly-rotated innominate bone in acetabular dysplasia causes increased the stress at the SIJ, and the changes induced by peri-acetabular osteotomy will aid decrease this stress.

## Methods

### Model creation and mesh generation

**Figure 1:**
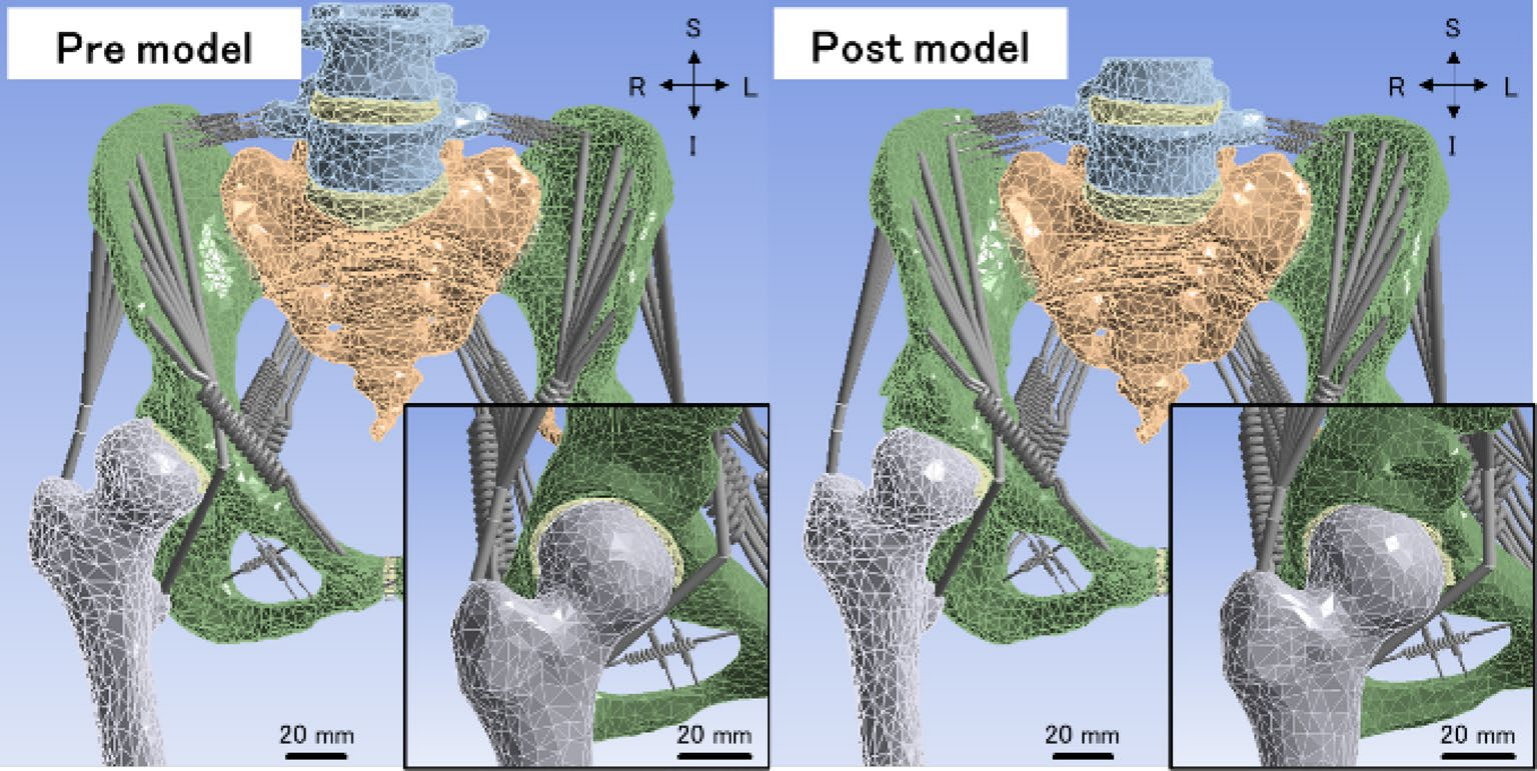
Finite element models of the preoperative (left) and postoperative (right) pelves of patient 1. The right is the surgical side. The insets are enlarged views of the right hip joints. The scale bar: 20 mm. I: inferior, L: left, R: right, S: superior. In the pre model, the acetabulum does not cover the femoral head sufficiently. In the post model, the acetabulum widens by the surgical intervention.

**Figure 2:**
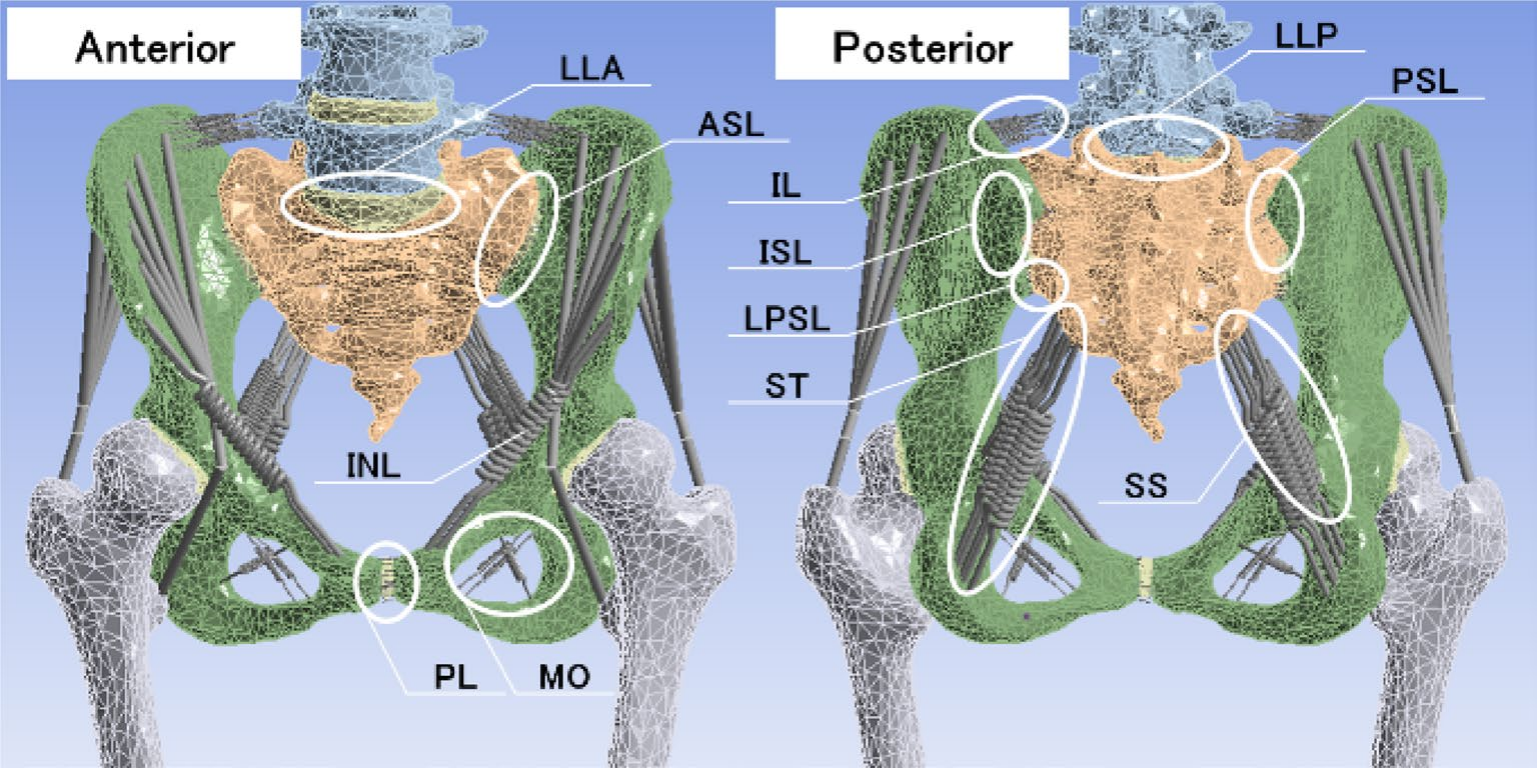
The positions and names of ligaments in a pelvic model of patient 1 with anterior (right) and posterior (left) view. The ligaments modelled are as follows: anterior longitudinal ligament (LLA), anterior sacroiliac ligament (ASL), inguinal ligament (INL), pubic ligament (PL), obturator membrane (MO), posterior longitudinal ligament (LLP), posterior sacroiliac ligament (PSL), iliolumbar ligament (IL), interosseous sacroiliac ligament (ISL), long posterior sacroiliac ligament (LPSL), sacrotuberous ligament (ST) and sacrospinous ligament (SS).

Finite element models of acetabular dysplasia pelves (Fig. 1) were created based on computed tomography (1-mm slice thickness) of preoperative (‘pre model’) and postoperative (‘post model’) pelves of four female patients (18–41 years old). All methods were carried out in accordance with relevant guidelines and regulations, and all patients provided the signed informed consent for use of the data. This study was approved by Institutional Review Board committee of Kanazawa Medical University. The bone and cartilage components were segmented in MECHANICAL FINDER ver. 10 (Research Center of Computational Mechanics, Inc., Tokyo, Japan) including the lumbar vertebra, the sacrum, both hip bones and proximal ends of both femora, as well as both SIJ cartilages, the pubic symphysis, both hip joint cartilages and the intervertebral disks. These geometries were modified and the SIJ cartilage was adapted to the bone shape in SpaceClaim 2021R1 (Cybernet Systems Co., Ltd., Tokyo, Japan). All models were then imported into ANSYS 2021R1 (Cybernet Systems Co., Ltd.). The twelve types of ligaments and two types of muscles surrounding the pelvis were modelled by a total of 210 spring elements^8^ and 20 beam elements, respectively (Fig. 2). The ligaments were defined in a way where they could respond only when they are subjected to tensile loads. Tensile forces of 720 N and 100 N were applied on the gluteus medius muscle and iliacus muscle on both sides, respectively^25^. Meshing was performed using tetrahedral elements consisting of ten nodes each (Supplementary Table S1). All figures of finite element models were displayed by ANSYS 2021R1 and modified by Microsoft PowerPoint on Microsoft 365.

### Material properties

The material properties used in this study were cited from previous studies^8,26,27^ (Table 1). All tissues were defined as being uniform and isotropic materials for simplification. The hyper-elastic material properties were defined using Mooney-Rivlin model, which is the strain energy density function W given by the following formula, as a complete non-compressional body.

$$W = C_{10} \left( {I_{1} - 3} \right) + C_{01} \left( {I_{2} - 3} \right) + C_{11} \left( {I_{1} - 3} \right)\left( {I_{2} - 3} \right).$$

**Table 1:** Material properties for finite element models. *C*_10_, *C*_01_ and *C*_11_ mean the parameter of Mooney-Rivlin model for hyper-elastic bodies.

Material	Young's modulus [MPa]	Poisson's ratio	*C*_10_ [MPa]	*C*_01_ [MPa]	*C*_11_ [MPa]
Bone^8,26,27^	11,000	0.2	–	–	–
Cartilage^8,27^	150	0.2	–	–	–
SIJ cartilage^8^	–	–	4.1	0.41	0
Symphysis cartilage^8^	–	–	0.1	0.45	0.6
Ligament^8^	350	–	–	–	–

Here, $C_{10}$ and $C_{01}$ are material constants, and $I_{1}$ and $I_{2}$ are the first and second invariant of the distortion.

### Loading and boundary conditions

Mimicking double-leg stance, 300 N and 600 N of loads were applied via the lowers ends of both femora, which were shortened to two thirds of the total length, and the base of the sacrum, respectively. The superior aspect of the second sacral spine and both femora were fixed in space. For contact type, all surfaces in contact were defined as “bonded”, which means the surfaces are fixed to each other.

### Measured parameters

**Figure 3:**
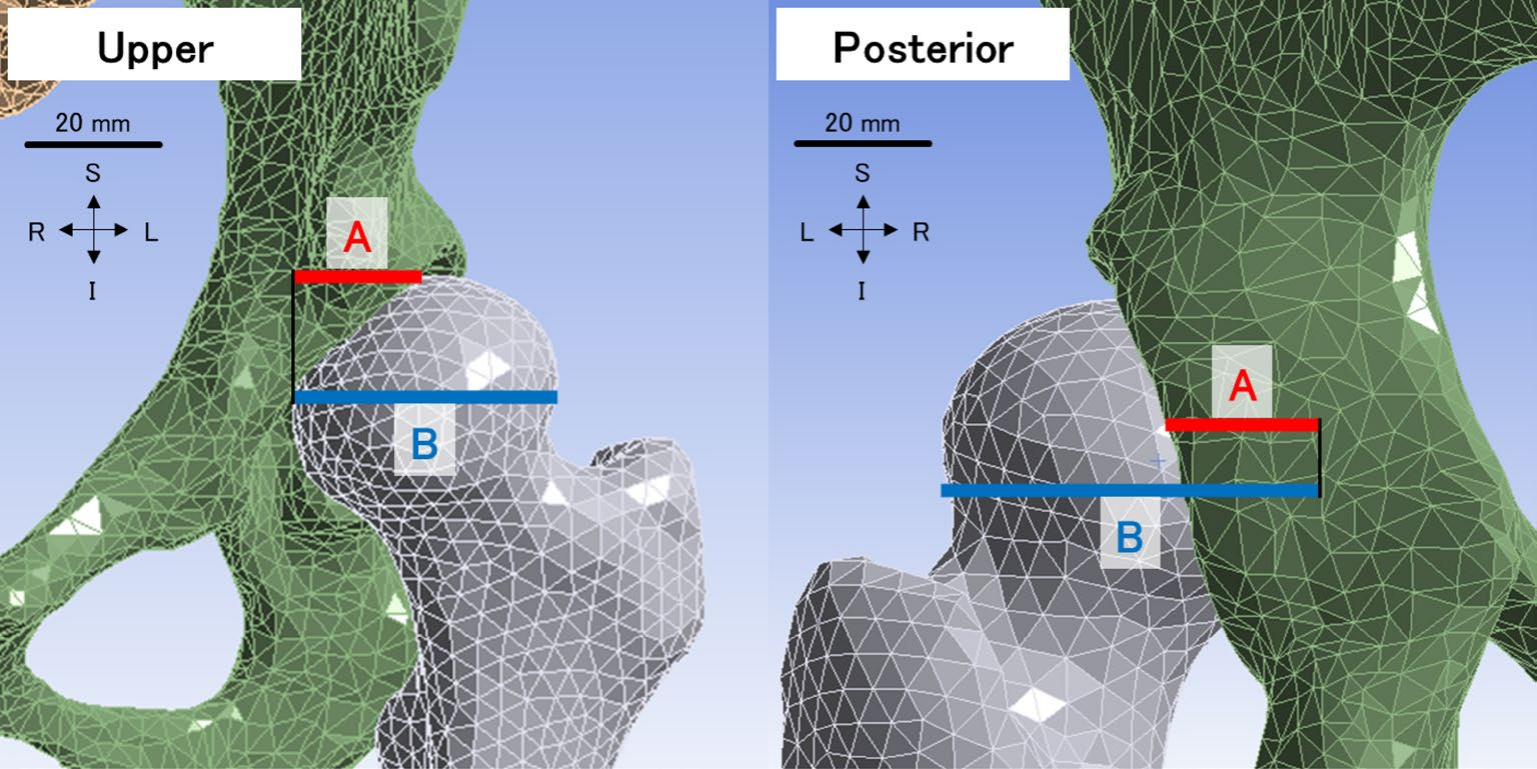
The upper (left) and posterior (right) acetabular head index (AHI) measurement positions with anterior (upper) and posterior (posterior) views. AHI is a ratio of A to B. A means a coverage of acetabulum on the top (upper) or center (posterior) of the femoral heads, and B means a diameter of the femoral head. The left femur and innominate bone are shown in grey and green, respectively. The scale bar: 20 mm. I: inferior, L: left, R: right, S: superior.

**Figure 4:**
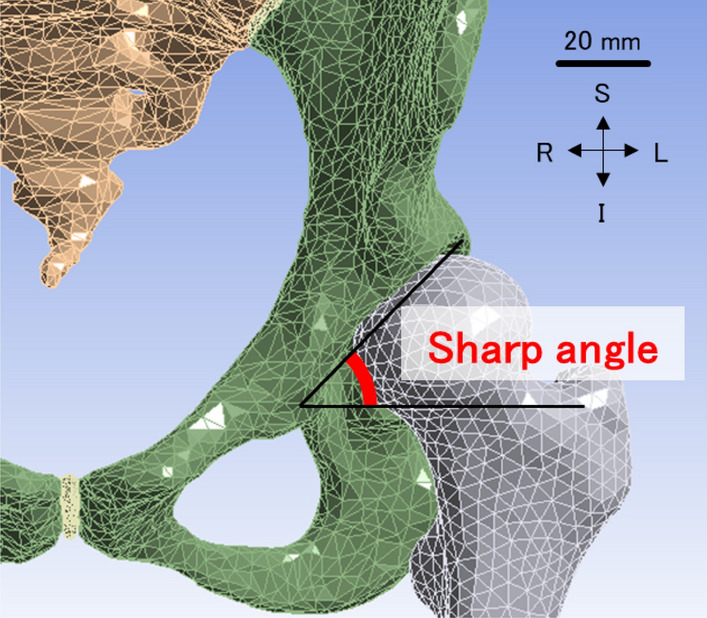
Sharp angle with an anterior view, measured between a horizontal line and a line from the tip of the pelvic tear drop to the lateral edge of the acetabular roof. The left femur and innominate bone are shown in grey and green, respectively. The scale bar: 20 mm. I: inferior, L: left, R: right, S: superior.

Acetabular head index (AHI, A/B) (Fig. 3) and sharp angle (Fig. 4) are indices for an assessment of acetabular dysplasia and are the ratio of acetabular coverage to the femoral head^28^ and the angle between the horizontal line and a line from the tip of the pelvic tear drop to the lateral edge of the acetabular roof^29^, respectively. Upper coverage (upper) and posterior coverage (posterior) of the femoral head diameter were measured. The resultant displacement of the pelves, equivalent (Von Mises) stress of the SIJ cartilages, the normal stress on lateral axes, the angles of rotation on the SIJs, the maximum elastic force of spring probes and acetabular head indices were investigated. Equivalent stress is a scalar value that is calculated from normal stresses and shear stresses without any distinction between tension and compression. Normal stress on lateral axes indicates the stress on the normal direction of the contact surfaces. Maximum elastic force of the spring elements was measured and summed for each of the ligaments as loads on ligaments.

## Results

### Acetabular head indices improved mainly in upper coverage of acetabulum

The AHIs on the side which underwent surgery improved − 1% to 22% (average 12.5%) in the upper area coverage and − 4% to 12% (average 5%), and in the posterior area coverage (Table 2). Coverage improved on both sites only in patient 1. Patients 2 and 4 improved in upper coverage, and patient 3 improved in the posterior coverage. The sharp angle on the side which underwent surgery improved from 51.8° to 42.5°. The average improvement was 9.3°.

**Table 2:** Measured acetabular head indices (AHI) of upper and posterior coverage and sharp angles on the surgical sides.

		Upper		Posterior		Sharp angle	Improvement (°)
		AHI (%)	Improvement (%)	AHI (%)	Improvement (%)		
Patient 1	Pre model	48	22	41	10	50°	8°
	Post model	70		51		42°	
Patient 2	Pre model	56	13	48	2	50°	9°
	Post model	69		50		41°	
Patient 3	Pre model	54	− 1	44	12	53°	9°
	Post model	53		56		44°	
Patient 4	Pre model	46	16	48	− 4	54°	11°
	Post model	62		45		43°	

Improve means an increase of AHI and a decrease of sharp angle from pre model to post model, respectively.

### Acetabular dysplasia pelves tended to rotate inward, called the ‘inflare’

**Figure 5:**
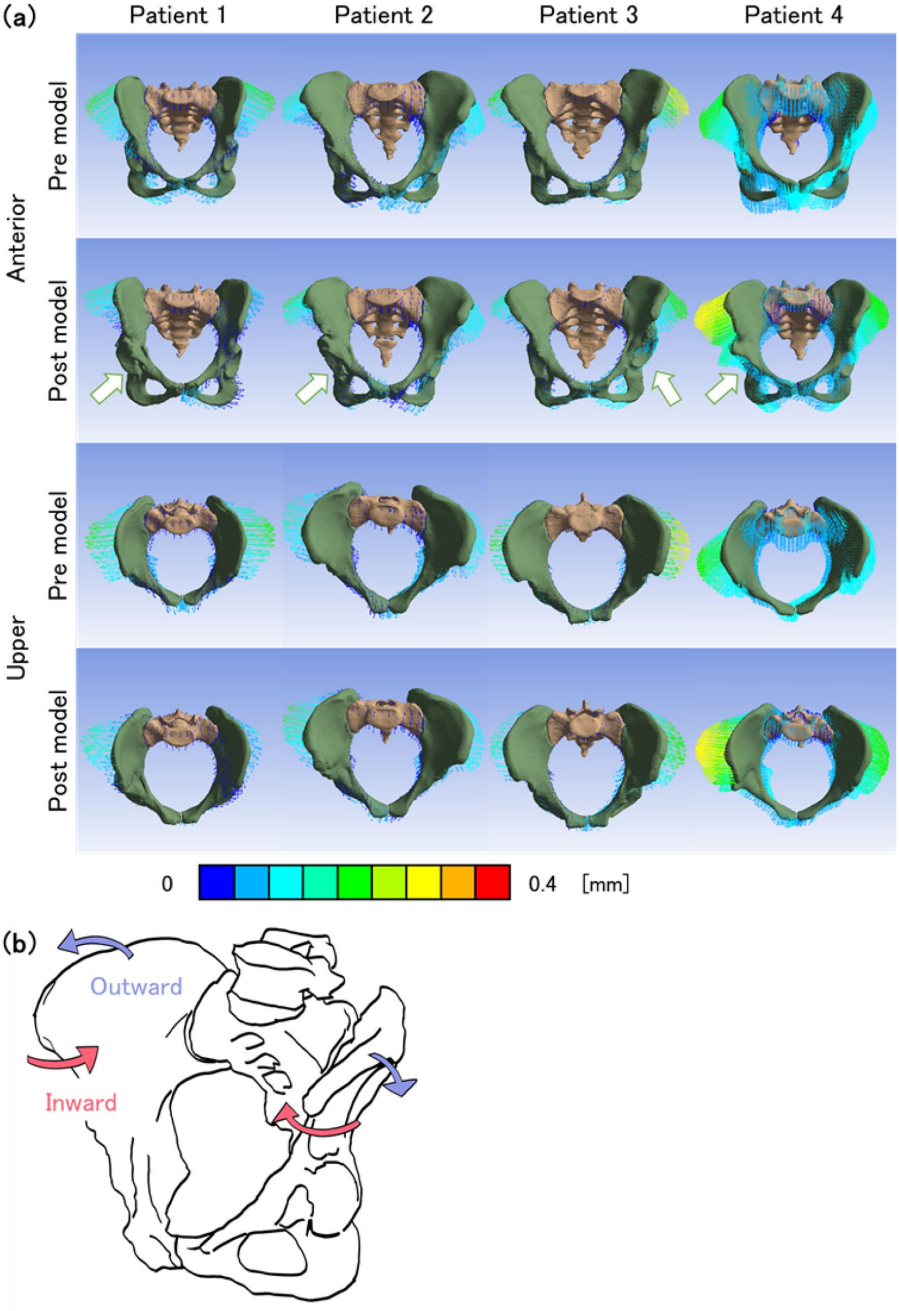
(**a**) Resultant displacement vector diagrams of the pelves on anterior (top 2 lines) and upper (bottom 2 lines) views. ‘Pre’ and ‘post’ reflect pre models and post models, respectively. The arrows indicate the sides of surgical intervention. (**b**) Scheme of pelvic deformation. The red and blue arrows indicate inward rotation (inflare) and outward rotation, respectively.

The displacement vector diagrams (Fig. 5a) of the pelves showed that the post models of all patients showed the innominate bones were deformed posteriorly. In the pre models derived from the patient datasets 1, 3 and 4, however, the innominate bones were deformed laterally to anteriorly. On the other hand, patient 2 in the pre models showed that the innominate bone was deformed posteriorly more than the post models. In the pre models of patients 1, 3 and 4 on acetabular dysplasia, the pelves rotated inward in the state of inflare as indicated by the red arrows in Fig. 5b. In the post models, the iliac crest was more rotated outward than in the pre models as indicated by the blue arrows in Fig. 5b. The maximum displacement on the iliac crest of surgical sides decreased by approximately 19% from pre models to post models in patients 1 and 3 which had a high improvement in posterior AHI more than 10%.

### Sacral nutation decreased when increased acetabular coverage

**Figure 6:**
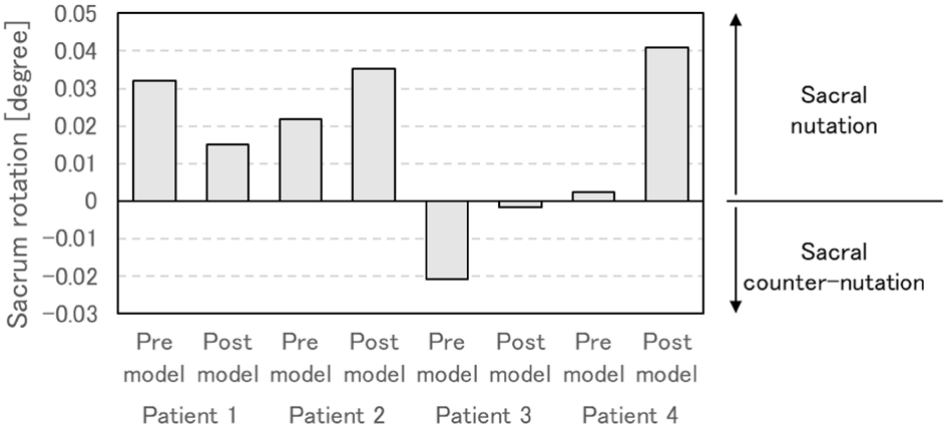
Degree of rotation at the sacroiliac joints on the sides of surgery in all patients, i.e., is the angle of rotation on the sacrum for ilium. The positive and negative values mean the nutation and counter-nutation, respectively.

In the surgical sides of post models, the rotation of SIJs decreased in patients 1 and 3, and increased in patients 2 and 4 compared to each pre models (Fig. 6). In addition, sacral nutation movement is decreased with posterior acetabular coverage and the overall improvement of the coverage led to counter-nutation (upper: r =  − 0.39, posterior: r =  − 0.68) (Supplementary Figure S1).

### The equivalent stress and compressive stress on sacroiliac joints cartilage decreased when increasing acetabular coverage

**Figure 7:**
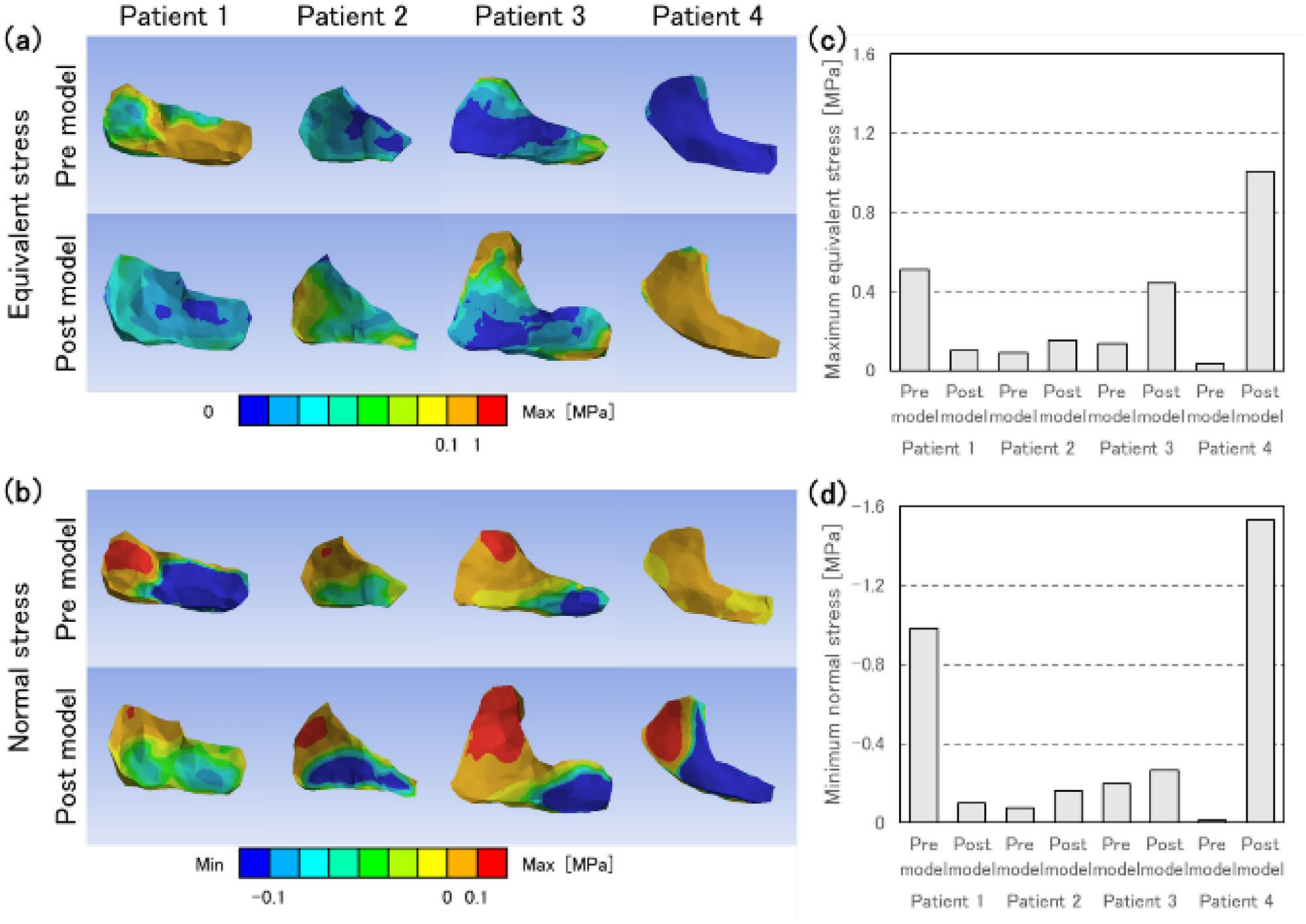
(**a**) Equivalent stress distribution and (**b**) normal stress distribution on the normal direction of the contact surfaces in sacroiliac joints on surgical sides. (**c**) Maximum equivalent stress and (**d**) minimum normal stress in sacroiliac joints on surgical sides.

The equivalent stress of SIJ cartilage decreased on the posterior regions in patient 1, and increased in the superior regions in all four cases (Fig. 7a). Maximum equivalent stress of SIJ cartilage ipsilateral to the surgical sides of pre models decreased by 80% in patient 1, while it increased by 67-2834% in the other cases compared to the post models (Fig. 7c). Normal stresses yielded that the SIJs were compressed on the inferior and extended on the superior regions (Fig. 7b). Minimum normal stress on the surgical sides decreased by 90% in patients comparing the pre models to the post models, and increased by 36–11,204% in the other cases (Fig. 7d). In patients 1 and 3, the posterior coverages were highly increased (10% and 12% increase), and the stresses decreased or slightly increased. Patients 2 remained the posterior coverage (2% increase) and patient 4 were decreased (4% decrease). These models showed slightly or dramatically increment. Thus, posterior acetabular coverage improvement reduced the maximum equivalent stress and minimum normal stress (equivalent stress: r = 0.26 (upper), 0.71 (posterior); normal stress: r = 0.13 (upper), 0.83 (posterior)) (Supplementary Figures S2 and S3)﻿.

### Loading of pelvic ligaments was reduced when acetabular coverage was increased

**Figure 8:**
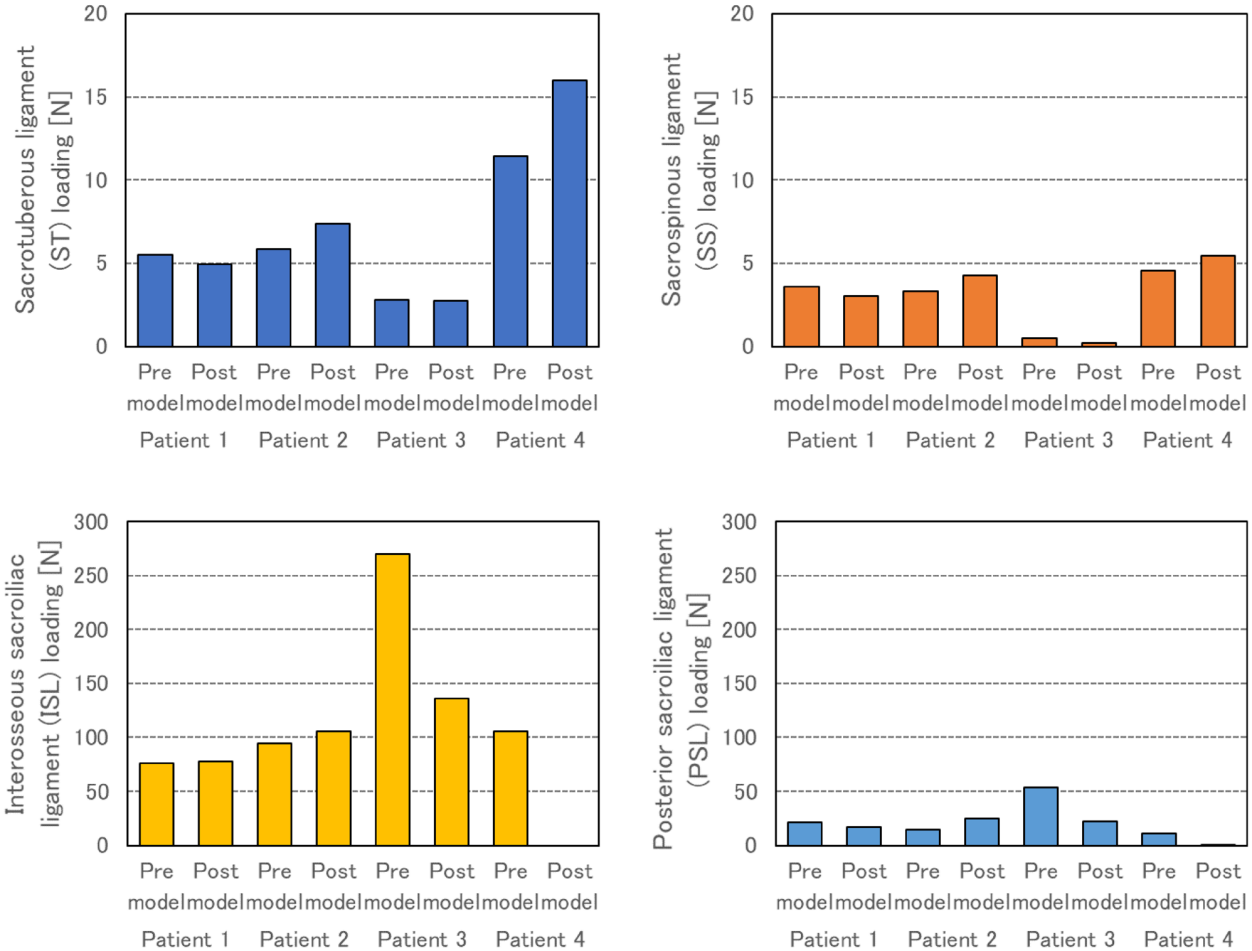
Sacrotuberous ligament (ST), sacrospinous ligament (SS), interosseous sacroiliac ligament (ISL) and posterior sacroiliac ligament (PSL) loading on the surgical sides of all patients.

The ligament loading on the sides of surgical intervention of the pre models compared to the post models decreased by approximately 36% in patients 1 and 3, and increased by 27% in patients 2 and 4. The change was mainly due to a decrease in posterior sacroiliac ligament (PSL) loading and an increase in interosseous sacroiliac ligament (ISL) loading (Fig. 8). In relation to the decrease in the ligament loading and the improvement of acetabular coverage, sacrotuberous ligaments (ST) and sacrospinous ligaments (SS) loading decreased with increased posterior coverage, and interosseous sacroiliac ligaments (ISL) and posterior sacroiliac ligaments (PSL) loading increased with increased upper coverage (upper: r =  − 0.19, − 0.08, 0.67, 0.70, posterior: r = 0.96, 0.89, 0.02, − 0.46, respectively) (Supplementary Figures S4, S5, S6 and S7)﻿.

## Discussion

To date, few studies have investigated the morpho-mechanical coupling between the hip joint and the SIJ under instances of physiological loading^30,31^. To our best knowledge, this is the first report investigating the effects of acetabular dysplasia on the stress environment of the SIJ. In a pelvis with acetabular dysplasia, the innominate bone rotates inward^5^, called in-flare. The morphological results of the preoperative models obtained from bone scanning also showed in-flare and similar results were observed. Following the surgical intervention, this deformation was restored to a non-pathological anatomy including a normal or out-flare state, except for patient 2 of this given study. The in-flare state seems to open the posterior part of the SIJ, thereby increasing the load on the posterior ligaments, and increasing the compressive load onto the superior part of the SIJ. On the other hand, in the out-flare state, the superior side of SIJs is thought to be opened and to be exposed to high tensile load. In patients 1, 3 and 4, the loading of posterior sacroiliac ligament (PSL) decreased by 22–91% from the preoperative to the postoperative state, indicating that the in-flare state changed to the out-flare state by peri-acetabular osteotomy for normalization of the acetabular bone coverage of the femoral head. Since the equivalent stress of the SIJ cartilage decreased only in patient 1, the equivalent stress state of the SIJ cartilage was determined by an increase in compressive loading due to the in-flare position, and an increase in tensile loading due to the out-flare position. The equivalent stress is considered to increase or decrease depending on the extent of rotation. However, the normal stress of the SIJ cartilage decreased only in patient 1 following the surgery, which was inconsistent with an increase in tensile loading due to out-flare. This may be explained as follows: the gluteus medius muscle pulls the iliac crest outward, therefore the ilium is pressed against the sacrum at the lower part of the SIJ to generate compressive force. In consequence, the tensile load should not increase at the anterior aspect of the SIJ even though out-flare was induced. In addition, since the compressive force at the SIJ tended to decrease with the improvement of the acetabular coverage, it was considered that the in-flare tended to result in lowered compressive loads. In this analytical model, the improvement of the posterior coverage was small, and the compressive load region tended to widen.

Some researchers suggest that the cause of pain originating from the SIJ is a consequence of overloading of the joint’s cartilaginous region and its stabilizing ligaments^13,32,33^. The lower part of the SIJ cartilaginous regions has been shown to be particularly stressed in patients with SIJ dysfunction^34^. At the same time, the SIJ has the role of transmitting the load from the trunk to the lower limbs in the sense of form and force closure, which requires compressive forces to fulfill this function^35^. Therefore, two possible pathways may exist to explain the stress environment at the SIJ due to acetabular dysplasia: relieved in-flare reduced the equivalent stress of the SIJ cartilage and ligament loading (patient 1); additional force closure due to the site of compressive loading may likewise increase stability (increased considerable stress) and decreased ligament loading (patient 3). In finite element analyses of the healthy pelvis, it has been reported that high equivalent stress is concentrated in the superior part of the SIJ cartilage^8,27^. In this given study, the postoperative model showed that following surgery, similar equivalent stress distribution was close to that of a healthy person. With surgery, the equivalent stress environment trended to become normal. At the same time, the pre models showed relatively low equivalent stress in the superior region of the SIJ cartilage. It has been observed in Japan that patients with acetabular dysplasia secondary progress the hip OA^36^, and Asada et al. have reported that the volume of vacuum phenomena in the SIJ, which indicate the SIJ degeneration, was significantly larger in the hip OA and the vacuum areas were localized in the antero-superior region of the SIJ^18^. The abnormal low equivalent stress at the same part may relate to the SIJ degeneration in acetabular dysplasia pelvis. The high equivalent stress distribution in the lower part of the SIJ cartilage before surgery seemed to be characteristic of acetabular dysplasia. Compared with the report that patients with SIJ dysfunction were loaded at the same position^34^, it is suggested that acetabular dysplasia may impair the stress environment in the SIJ. Several reports exist on alterations in pelvis biomechanics following lumber and/or SIJ fixation and hip OA. Ivanov et al. investigated stress distribution of the pelvis with lumber fixation using finite element analysis, and showed that the equivalent stress and angular motion of the SIJ increased when compared to the state before surgical fixation^37^. Ha et al. have reported that SIJ degeneration was observed in 75% of patients on X-rays five years after posterior fusion of the lumbar spine^38^. It has been reported that hip OA significantly progresses 12 months in patients following surgical lumbar or SIJ fusion^31^. Vice versa, it has been reported that SIJ degeneration progresses in patients with hip OA before hip replacement surgery^18^. It can be concluded based on these published findings that spine conditions such as lumbar and SIJ fixation and acetabular conditions such as hip OA may interact with each other. These reports show that surgical interventions and hip OA which cause loss of joint movement may have a negative effect on adjacent joints. In this study, the results showed that surgery to improve acetabular dysplasia had a positive effect on improving the stress environment of the SIJ. However, one research report indicates that SIJ fixation imparted little change of stress at the hip joint^30^. The spine may affect the hip joint more than the condition of the SIJ.

Various indices such as center–edge angle (CEA)^39^, acetabular roof angle (ARO)^40^, acetabular head index (AHI)^28^ and sharp angle^29^ are used clinically to assess the extent of acetabular dysplasia. Physicians diagnose acetabular dysplasia at a CEA of 20° or less^41^. It should be noted that in this study, AHI and sharp angle were investigated, but this meant upper and posterior coverage of acetabulum. The improvement of the coverage of the posterior acetabulum was considered to lead to counter-nutation, which is assumed to stabilize the pelvis and to reduce the equivalent stress on the SIJs and the load on the main ligaments around the pelvis. The SS, ST, and ASL were suggested to have a defined function of limiting the nutation movement^42,43^, and a decrease in these ligaments’ loading can be observed with the improved posterior coverage of the acetabulum, indicating that nutation movement weakens. The results were similar to those of the counter-nutation induction obtained from the measurement of the rotation of the SIJ. The decrease in displacement at the iliac crest after surgery indicated that the load from the femora could be stable be stably transmitted to the innominate bone. The improvement of the posterior coverage of the acetabulum has been suggested to normalize the load transmission on the pelves and to improve the stress environment of SIJs. However, it has been reported that an over-improvement of the posterior coverage limits the range-of-motion of the hip joint^44^. The change in the stress environment of the pelvis due to the improvement of the covering therefore seems to be limited.

This study has a number of limitations. First, the conclusions were derived from a small sample size which was available. In addition, only female patients were investigated. Female pelves have a high sacral slope which means SIJs are easily exposed to shearing stress. Since the shearing force is applied more to female than to male^45,46^, the load on the SIJ due to hip joint disease and the change in load on the SIJ after surgery may be more significant for females. Secondly, only a subset of anatomical structures has been modeled, including the most relevant osseous, cartilaginous, ligamentous and muscular components. Further refinement of the given models may help gain more insight into the variations in load distribution related to surgery of acetabular dysplasia. Another limitation is related to the choice of the material models (isotropic, homogeneous) and testing conditions (quasi-static). Furthermore, the tissues around the hip joint were composed of deformable cartilage and two major muscles and did not design ligaments nor joint slip. The actual hip joint slides in the joint capsule during exercise, but this analysis did not take this into consideration. However, the analytical condition of this given simulation was only in standing position, where the bones did not move enough to perform intracapsular movement in the hip joint, so only compressive force transmission occurred. Therefore, this model seemed to reproduce the stress environment in the pelvis during standing.

## Conclusions

CT datasets of pelves of patients with acetabular dysplasia undergoing surgery were modelled to assess stress distribution in double-leg stance in a preoperative and postoperative condition. Comparing the pre- and postoperative findings, the preoperative innominate bones were rotated inward, called ‘inflare’. Improving the posterior coverage of the acetabulum relieved this rotation and the innominate bones were deformed laterally to anteriorly. This reduced the equivalent stress, compressive stress and ligament loading at the SIJ, suggesting that acetabular morphology may affect the SIJ stress environment.

## Supplementary Information

Supplementary Information.

## Data Availability

The datasets analyzed during this given study are available from the corresponding author on reasonable request.
