# Supplementary material for: A patient-cohort study of numerical analysis on sacroiliac joint stress distribution in pre- and post-operative hip dysplasia
Source: Sci Rep. 2022 Aug 25;12:14500. doi: 10.1038/s41598-022-18752-1 (PMC9411127; doi:10.1038/s41598-022-18752-1)
Supplement: Supplementary file 1 — Supplementary Information. [file 41598_2022_18752_MOESM1_ESM.docx]

Supplementary Information for:

**A patient-cohort study of numerical analysis on sacroiliac joint stress distribution**

**in pre- and post-operative hip dysplasia**

Ryota Toyohara^1*^, Ayumi Kaneuji^2^, Noriyuki Takano^3^, Daisuke Kurosawa^4^, Niels Hammer^5,6,7^, Toshiro Ohashi^8^

1 Graduate School of Engineering, Hokkaido University, Japan

2 Department of Orthopedic Surgery, Kanazawa Medical University, Japan

3 Integrated Technology Research Center of Medical Science and Engineering, Kanazawa Institute of Technology, Japan

4 Department of Orthopedic Surgery / Japan Sacroiliac Joint and Low Back Pain Center, JCHO Sendai Hospital, Japan

5 Division of Clinical and Macroscopic Anatomy, Gottfried Schatz Research Center, Medical University of Graz, Austria

6 Department of Orthopedic and Trauma Surgery, University of Leipzig, Germany

7 Fraunhofer Institute for Machine Tools and Forming Technology (IWU), Medical Branch, Germany.

8 Faculty of Engineering, Hokkaido University, Japan

*Correspondence: toyohara.rt@gmail.com

**Supplementary Table S1**

|  | | Number of nodes | Number of elements | Average element quality | Surgical side |
| --- | --- | --- | --- | --- | --- |
| Patient 1 | Pre model | 253,909 | 144,502 | 0.72 | Right |
|  | Post model | 227,920 | 128,871 | 0.72 |  |
| Patient 2 | Pre model | 242,013 | 138,004 | 0.71 | Right |
|  | Post model | 253,002 | 144,887 | 0.72 |  |
| Patient 3 | Pre model | 249,850 | 142,448 | 0.71 | Left |
|  | Post model | 242,042 | 138,512 | 0.72 |  |
| Patient 4 | Pre model | 219,098 | 124,072 | 0.71 | Right |
|  | Post model | 223,523 | 127,029 | 0.71 |  |

Number of nodes and elements and average element quality of each finite element models. Surgical sides of all patients. The average element quality is a composite quality metric given in the ranges between 0 and 1.


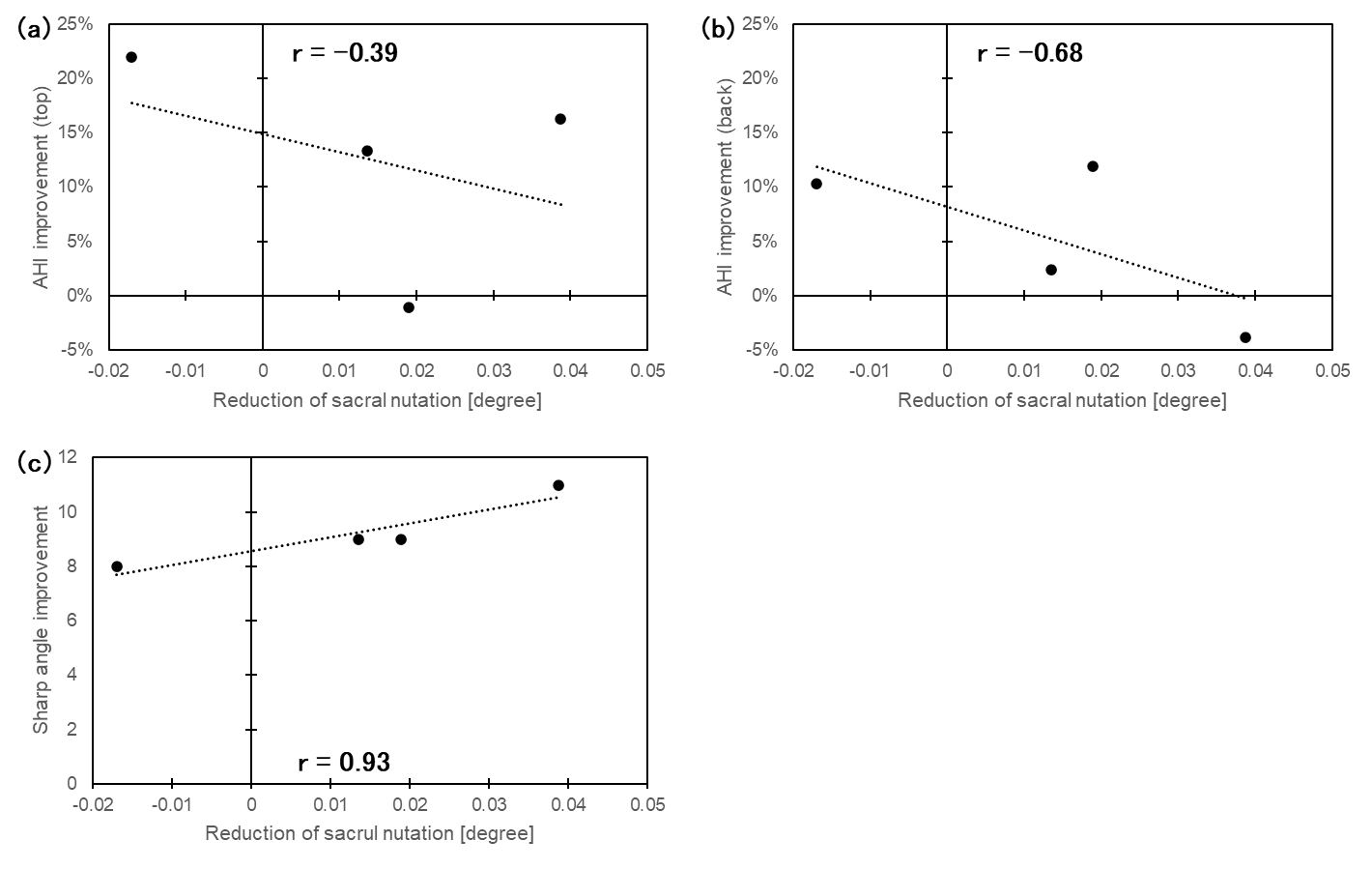
**Supplementary Figure S1: Sacral nutation**

The scatter plots of reduction of sacral nutation on the surgical sides vs. improvement of acetabular head index (a) on upper coverage and (b) on posterior coverage, and (c) vs. improvement of sharp angle.

**Supplementary Figure S2: Maximum equivalent stress**


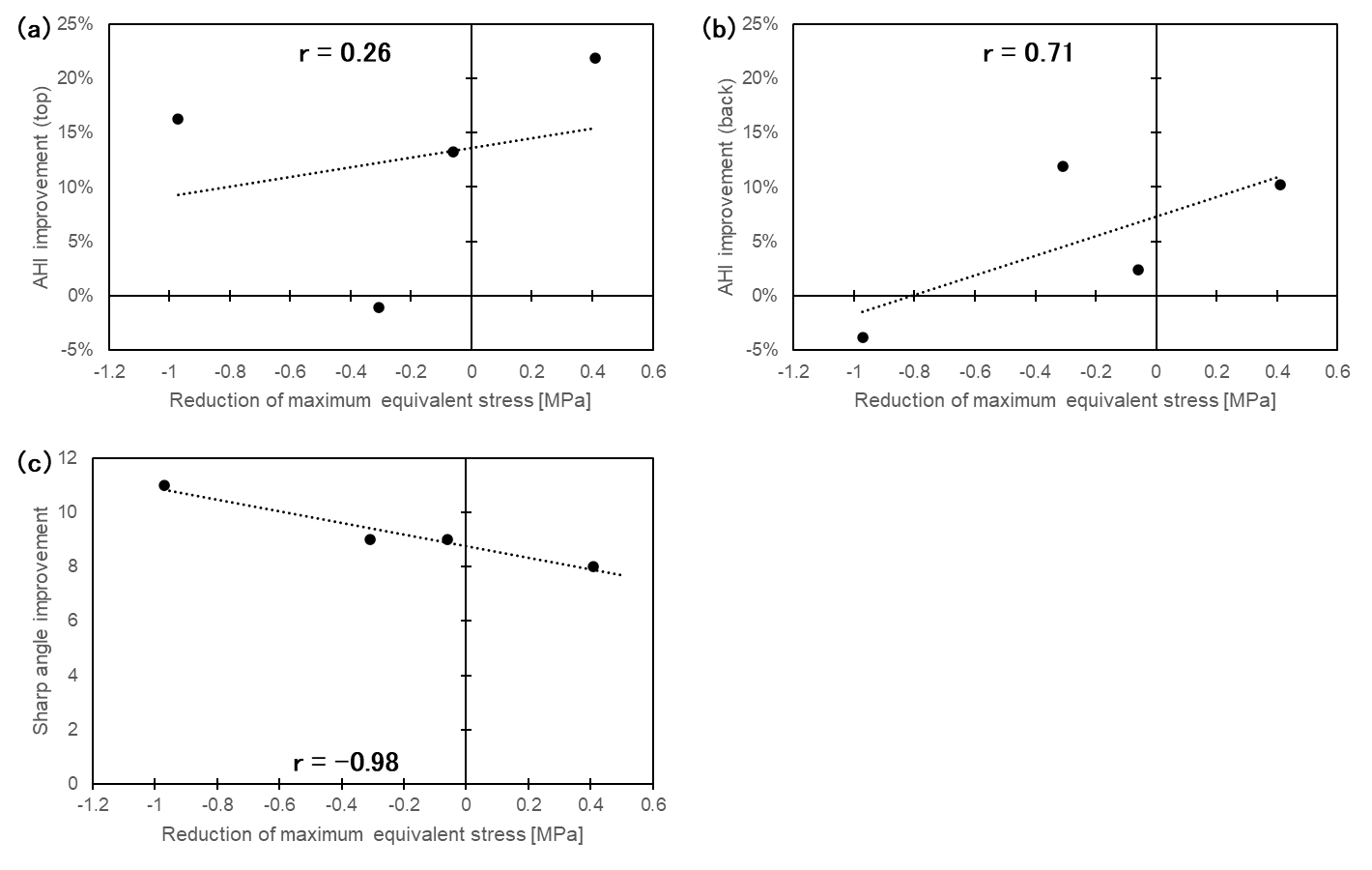


The scatter plots of reduction of maximum equivalent stress in the sacroiliac joint on the surgical sides vs. improvement of acetabular head index (a) on upper coverage and (b) on posterior coverage, and (c) vs. improvement of sharp angle.

**Supplementary Figure S3: Minimum normal stress**


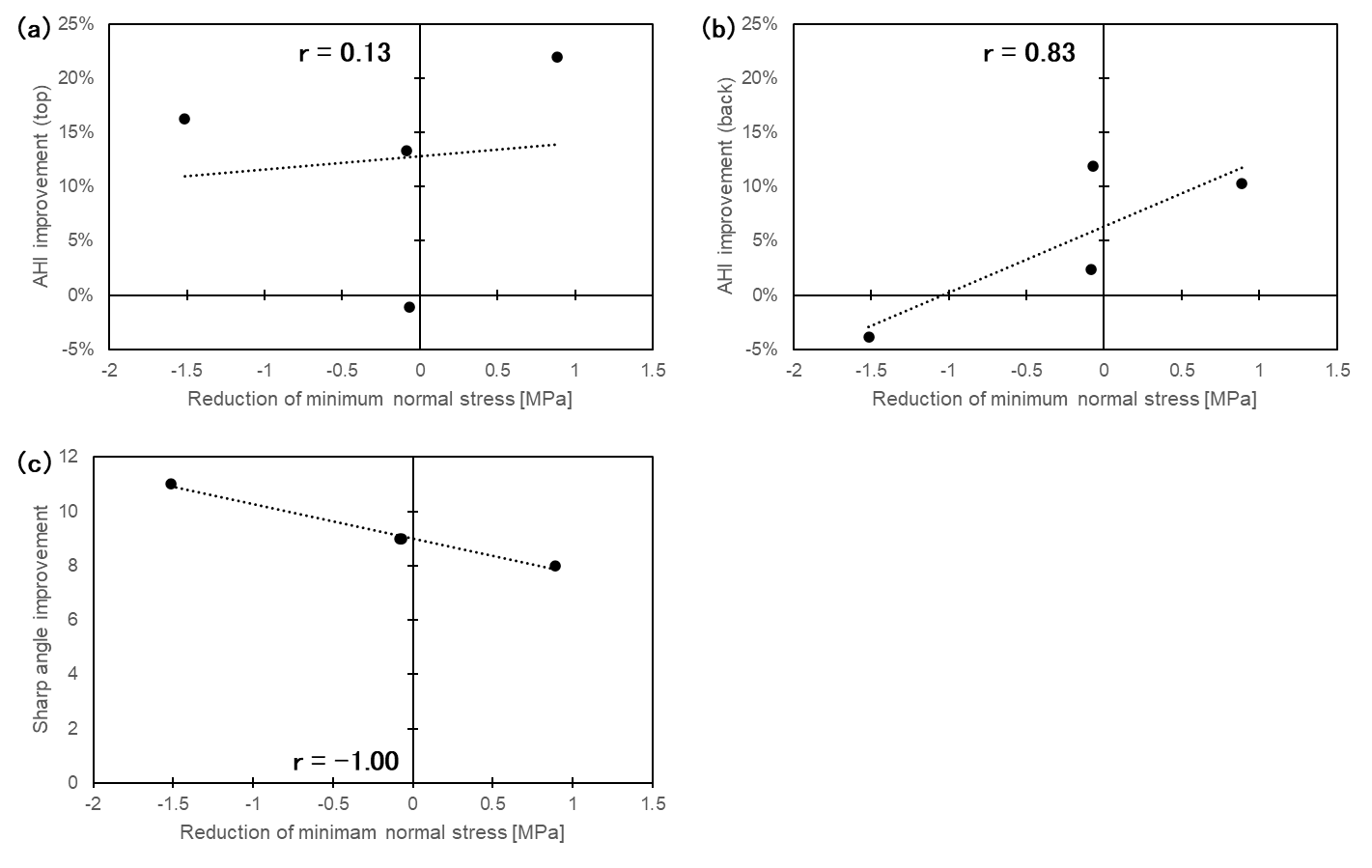


The scatter plots of reduction of minimum normal stress in the sacroiliac joint on the surgical sides vs. improvement of acetabular head index (a) on upper coverage and (b) on posterior coverage, and (c) vs. improvement of sharp angle.


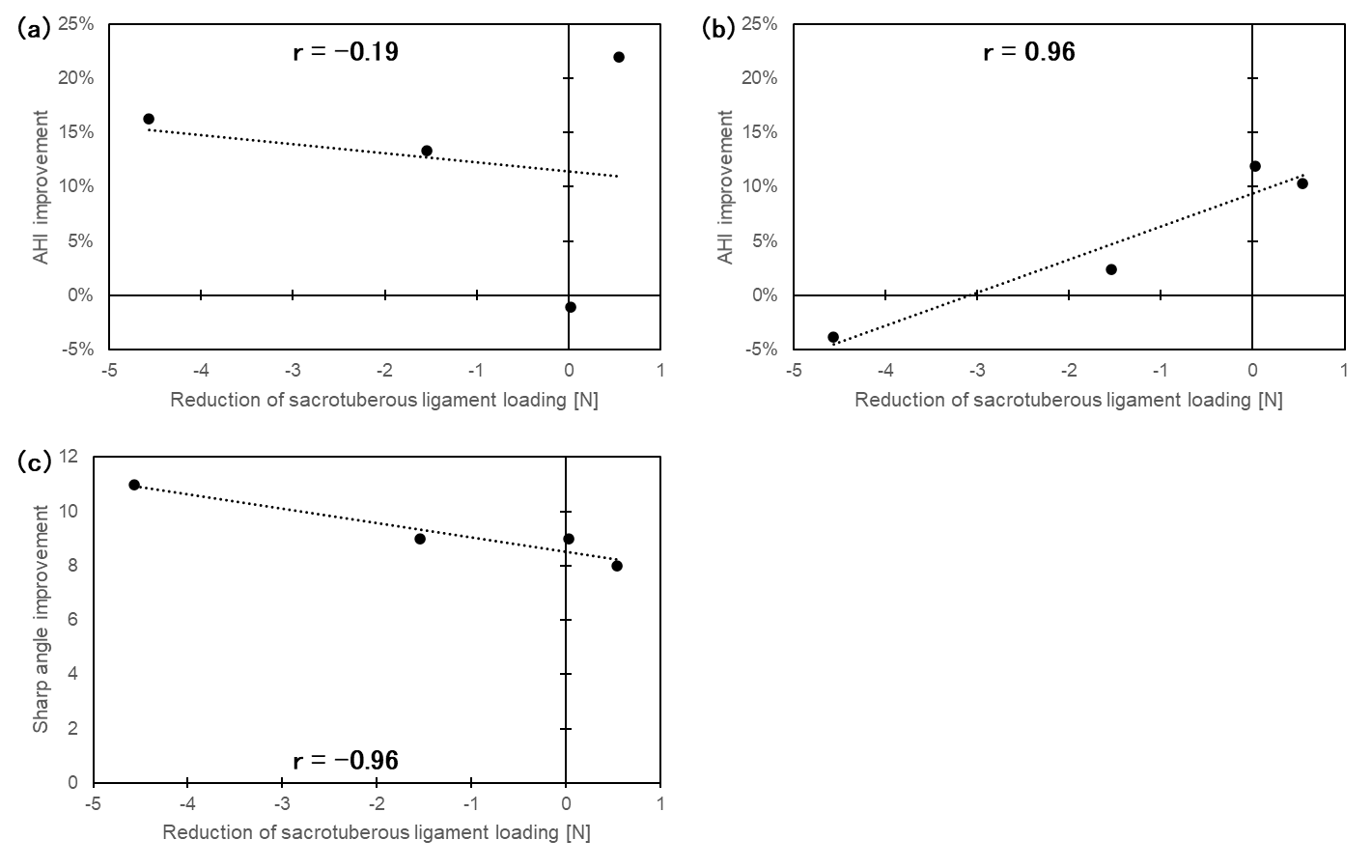
**Supplementary Figure S4: Sacrotuberous ligament (ST) loading**

The scatter plots of reduction of sacrotuberous ligament (ST) loading on the surgical sides vs. improvement of acetabular head index (a) on upper coverage and (b) on posterior coverage, and (c) vs. improvement of sharp angle.

**
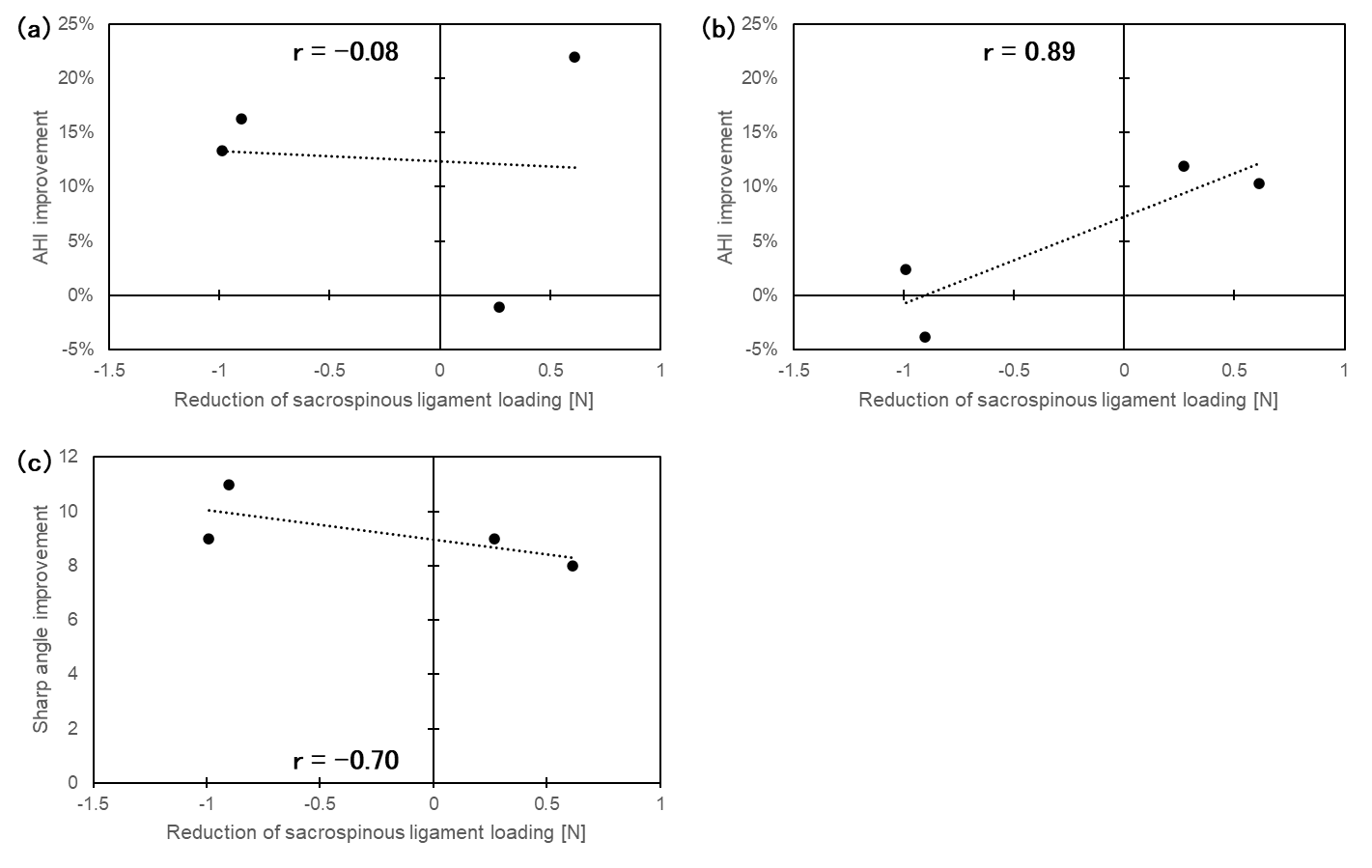
Supplementary Figure S5: Sacrospinous ligament (SS) loading**

The scatter plots of reduction of sacrospinous ligament (SS) loading on the surgical sides vs. improvement of acetabular head index (a) on upper coverage and (b) on posterior coverage, and (c) vs. improvement of sharp angle.


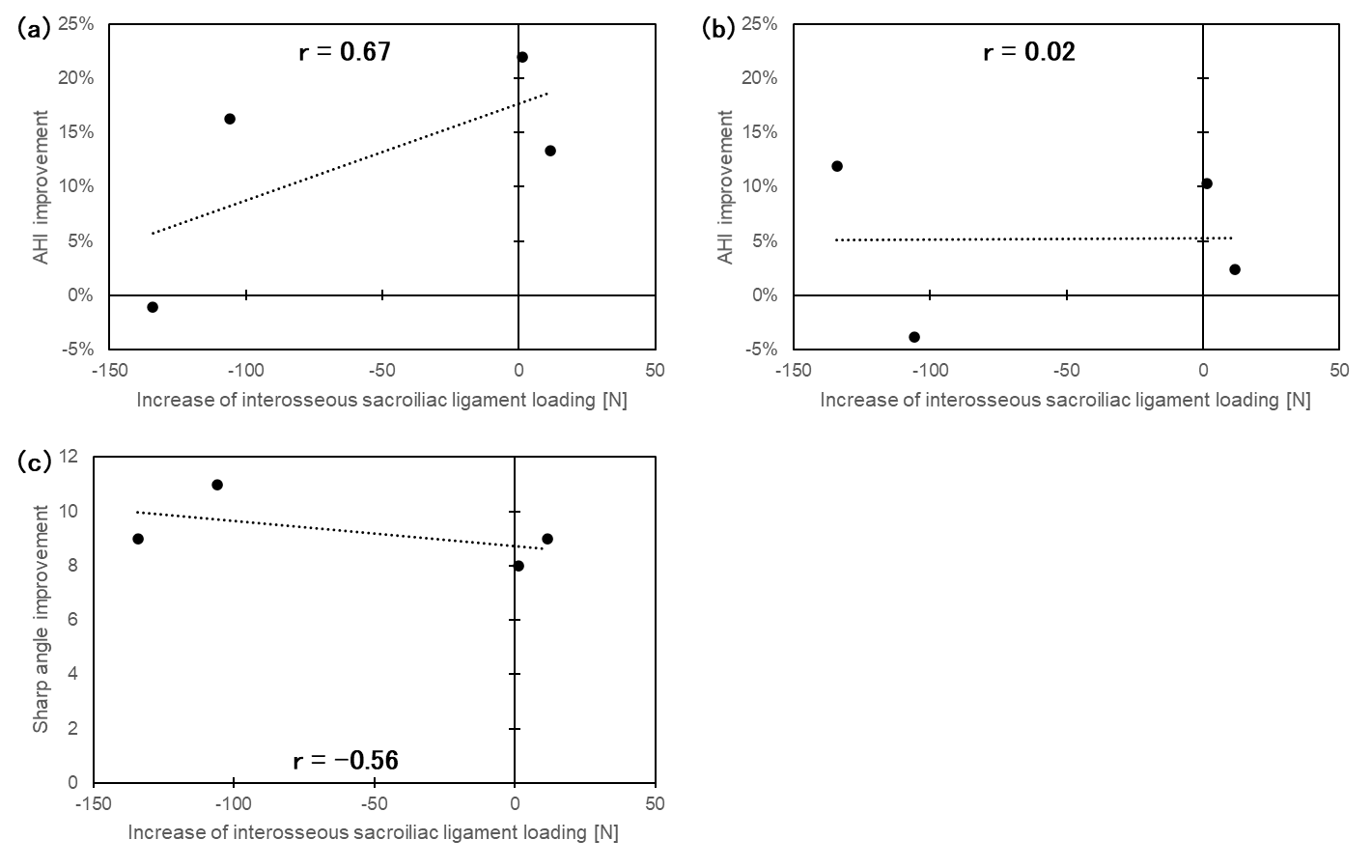
**Supplementary Figure S6: Interosseous sacroiliac ligament (ISL) loading**

The scatter plots of increase of interosseous sacroiliac ligament (ISL) loading on the surgical sides vs. improvement of acetabular head index (a) on upper coverage and (b) on posterior coverage, and (c) vs. improvement of sharp angle.


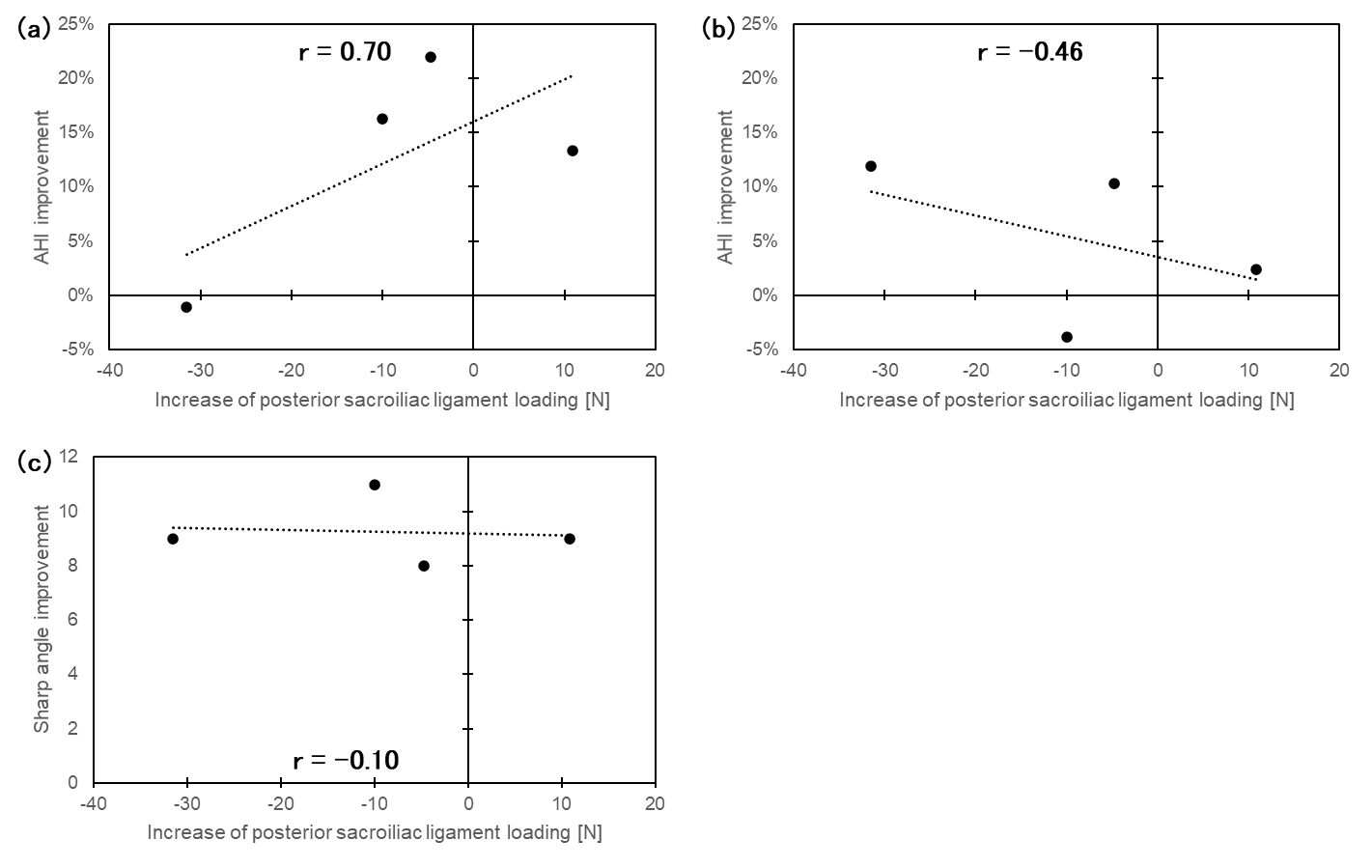
**Supplementary Figure S7: Posterior sacroiliac ligament (PSL) loading**

The scatter plots of increase of posterior sacroiliac ligament (PSL) loading on the surgical sides vs. improvement of acetabular head index (a) on upper coverage and (b) on posterior coverage, and (c) vs. improvement of sharp angle.
